# Absent Pulmonary Artery Presenting as High-Altitude Pulmonary Edema

**DOI:** 10.5811/cpcem.2022.9.57506

**Published:** 2022-11-04

**Authors:** Douglas Dillon, Austin T. Smith

**Affiliations:** Intermountain Healthcare, Department of Emergency Medicine, Park City, Utah

**Keywords:** absent pulmonary artery, high altitude pulmonary edema, environmental emergencies, shortness of breath, dyspnea

## Abstract

**Case Presentation:**

A 22-year-old male with no known past medical history presented to our emergency department complaining of difficulty breathing. A plain film chest radiograph revealed findings consistent with a tension pneumothorax.

**Discussion:**

However, due to physical examination findings inconsistent with the imaging report, a computed tomography of the chest was ordered which revealed an absent right pulmonary artery.

The patient was ultimately treated for high altitude pulmonary edema and discharged on nifedipine and supplementary oxygen.

## CASE PRESENTATION

A 22-year-old male presented to our emergency department (ED) complaining of difficulty breathing. The patient was visiting from sea level and driving a snowmobile at an elevation just above 9,000 feet when he became short of breath. He denied any trauma. He presented to our ED (elevation 5,412 feet) with improvement but ongoing dyspnea. On arrival to the ED, the patient had a heart rate of 113 beats per minute, and he was breathing 40–45 times per minute with an oxygen saturation of 89% on 15 liters nonrebreather mask. His pulmonary exam was significant for bilateral breath sounds with wheezing that was more prominent on the left side.

**Image 1:**
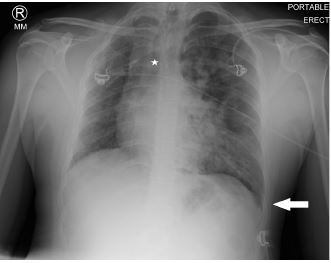
An anteroposterior plain film of the chest revealing a deviated trachea (star) and a deep sulcus sign (arrow), in addition to fluffy infiltrates in the left lung fields.

**Image 2:**
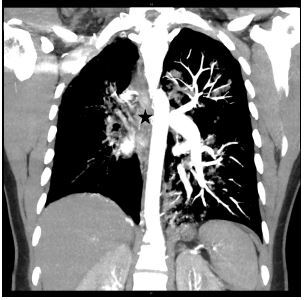
A coronal view of a contrast-enhanced computed tomography angiography revealing an absent right pulmonary artery (star) and hypoplastic right lung.

**Image 3:**
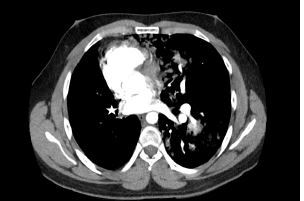
An axial view of a contrast-enhanced computed tomography angiography revealing an absent right pulmonary artery (star).

A plain film of the chest was ordered, which was interpreted by radiology as probable left-sided tension pneumothorax (Image 1). However, the treating physician thought that pulmonary edema was more likely based on real-time interpretation of this image along with physical exam findings. Due to an inconsistency between the clinical presentation and imaging report, a contrast-enhanced computed tomography angiography was ordered, which revealed an absent right pulmonary artery and hypoplastic right lung (Images 2, 3).

The patient was transferred to a facility at a lower elevation and discharged five days later on nifedipine and supplementary oxygen. The final diagnosis was high-altitude pulmonary edema (HAPE) and absent right pulmonary artery.

## DISCUSSION

Absent pulmonary artery is a rare condition, occurring in approximately 1 in 200,000 adults^1^ Usually it is accompanied by other cardiovascular abnormalities, but it can be isolated.^2^ Due to the nature of the embryologic development, it most commonly occurs on the right side.^3^ Presentation and diagnosis vary but is typically found while evaluating for pulmonary hypertension, suspected structural lung disease, or cardiac evaluations. Interestingly, HAPE is seen in approximately 10% of patients with an absent pulmonary artery.^3^–^6^ This is thought to be caused by development of pulmonary hypertension secondary to altitude and exercise at higher elevation.^5^

**CPC-EM Capsule**

What do we already know about this clinical entity?

*Absent pulmonary artery is a rare condition frequently diagnosed when evaluating for causes of dyspnea. Patients with this condition are particularly susceptible to high altitude pulmonary edema*.

What is the major impact of the image(s)?

*It is important for clinicians to ensure that a radiologic findings are consistent with clinical conditions; if not, more investigation should be sought*.

How might this improve emergency medicine practice?

*This case is an excellent example of the importance of clinical correlation and not anchoring on a diagnosis. Had the clinician relied upon the radiologic interpretation, this patient could have had a catastrophic outcome*.
